# High adherence to malaria treatment: promising results of an adherence study in South Kivu, Democratic Republic of the Congo

**DOI:** 10.1186/s12936-015-0933-7

**Published:** 2015-10-19

**Authors:** Sibylle Gerstl, Alexis Namagana, Liliana Palacios, Franklin Mweshi, Stella Aprile, Angeles Lima

**Affiliations:** Médecins Sans Frontières (MSF), Barcelona, Spain; MSF, Shabunda, South Kivu Democratic Republic of the Congo; Governmental Reference Hospital, Shabunda, South Kivu Democratic Republic of the Congo; MSF, Bukavu, South Kivu Democratic Republic of the Congo

**Keywords:** Malaria, Adherence, Compliance, Emergency setting, Conflict, Morbidity, Mortality, Treatment, Artemisinin-based combination therapy/ACT, Democratic Republic of the Congo

## Abstract

**Background:**

In resource-poor settings, treatment adherence is a major determinant of response to anti-malarial drugs as most are taken at home without medical supervision. Evidence on adherence to artemisinin-based combination therapy (ACT) is limited. The study aimed to measure adherence and identify reasons for non-adherence to a 3-day, fixed-dose combination (FDC) of artesunate–amodiaquine (ASAQ), the first-line treatment for uncomplicated malaria in the Médecins Sans Frontières project in the Shabunda Health Zone, South Kivu, Democratic Republic of Congo, a highly malarious and conflict-affected area.

**Methods:**

The study took place in the health centres/outpatient departments of the Shabunda general hospital, the quarter Mbangayo, and participant households. Patients prescribed FDC ASAQ were visited at home on the day after their regimen finished and asked to complete an adherence questionnaire. Patients/caretakers were also interviewed when exiting the outpatient department to understand their attitude towards FDC ASAQ and assess the quality of the prescribing process.

**Results:**

148 patients/caretakers completed the adherence questionnaire: 11.5 % (17/148, 95 % CI 7–17) had ≥1 tablet left at the time of the home visit and were defined as certainly non-adherent; 13.5 % (20/148, 95 % CI 8–19) were probably non-adherent; thus total non-adherence was 25.0 % (37/148, 95 % CI 18–32). 75 % (111/148, 95 % CI 68–82) were defined as probably adherent. In exit interviews, 87.5 % (105/120) knew they had malaria or could name the correct signs/symptoms. 89 % (107/120) could identify FDC ASAQ as anti-malarials among all tablets given and correctly repeat the intake instructions given at the outpatient department.

**Conclusions:**

This is the first study to assess adherence to an FDC of ACT under real treatment conditions in a context of high instability. High quality prescribing of anti-malarials at health centre level and patient adherence to the correct intake of ACT were possible in this setting. Adherence to treatment regimen requires careful and constant monitoring which might be better guaranteed at health centre rather than community level. It could, nevertheless, be a precondition to the successful introduction of home- or community based management of malaria.

## Background

Malaria, despite being treatable, remains a major killer in many low-income countries, with around half the world’s population at risk from the disease. Approximately 627,000 deaths occur every year, 90 % in sub-Saharan Africa and 85 % in children under 5 years of age [1, 2]. In addition to preventive measures, prompt and accurate diagnosis (through microscopy or rapid-diagnostic testing) followed by treatment with an artemisinin-based combination therapy (ACT) is the current main malaria control strategy. ACT has been adopted as national policy for first-line treatment in 79 of the 88 countries where *Plasmodium falciparum* is endemic [2]. ACT, however, should not be considered the definitive solution to the problem of anti-malarial resistance and correct and effective treatment of malaria presents enormous challenges, especially in sub-Saharan Africa [3]. Incorrect treatment intake and inadequate drug dosing increases drug pressure and thus the risk of developing resistance [4]. Worryingly, there are already concerns that ACT efficacy has declined in some parts of Asia [5, 6].

The correct use of a treatment involves correct diagnosis, prescription, dosage, and adherence. Factors that influence patient adherence include frequency of dosing, number of pills, duration of treatment, side-effects, cost of treatment, household income, concomitant treatment intake, education level, sufficient explanation of the importance of full-course adherence by the treatment prescriber, attitudes towards the sharing and saving of medications, and caregivers’ perception of the severity of the illness [7–14]. Only some of these factors can be directly influenced by treatment providers, such as ensuring complete and clear explanations on how to take the treatment and limiting the number of treatment schedules per patient.

Adherence to treatment is a major determinant of the response to anti-malarial drugs, especially for remote and unstable settings where home-based or community-based programmes for managing malaria might be one of the few strategies able to overcome barriers to accessing health care [15, 16]. Adherence should be regularly assessed in order to ensure that treatment strategies remain effective and to give the best chance of a positive treatment outcome. Such evaluations should be used as practical tools, allowing for appropriate action to improve patient adherence if indicated. However, the evidence on adherence to ACT is limited [17] and there is no consensual standard for what constitutes adequate adherence [18].

Fixed-dose combinations (FDC) might improve adherence compared with co-blistered drugs [19–21]. With the artesunate–amodiaquine fixed-dose combination (FDC ASAQ), adults take just two tablets per day compared with eight in the co-blister formulation [22]. Moreover, patients cannot separate the two drugs, thus avoiding monotherapy and preventing the development of drug resistance. Only two studies have evaluated FDC ASAQ, with reported adherences of 83 % in Madagascar and 90 % in Benin; however, in both studies, patients were expecting a home visit at the end of the treatment course, which might have led to improved treatment intake [23, 24]. In a study carried out by Médecins Sans Frontières (MSF) in 2009 in Boguila, Central African Republic, adherence to an FDC of ACT (Coartem™) was just 60.8 % [25].

In accordance with the national protocol of the Programme Nationale de Lutte contre le Paludisme (National Malaria Programme) of the Democratic Republic of the Congo (DRC), FDC ASAQ is the first-line malaria treatment used in the MSF project in the Shabunda Health Zone, South Kivu, DRC. All malaria cases are confirmed with a rapid diagnostic test (RDT HRP2 SD Bioline^®^) or microscopy at hospital level when indicated. Malaria is holoendemic (perennial and intense transmission) in Shabunda Health Zone, with the highest number of cases occurring between September and December. In January–October 2013, malaria cases in MSF-supported health facilities in Shabunda Health Zone accounted for 31.4 % of consultations in the outpatient department (OPD) (31,630/100,579); 48.4 % (15,313 cases) were in children aged under 5 years. Uncomplicated malaria was the principal cause of morbidity and the main cause for admission to a reference hospital (36.4 %, 3487/9586) (MSF, internal source, IPD-OPD database, 2013).

No adherence study has previously been carried out in the MSF project in the Shabunda Health Zone. In this study, the aim was to measure adherence to the currently used FDC ASAQ given over 3 days to treat uncomplicated malaria in a highly malarious and conflict-affected region and to identify the reasons for non-adherence.

## Methods

### Study area, period and population

Shabunda town, population approximately 25,000, is the capital of Shabunda Health Zone, a region comprising 20 health areas (“aires de santé”) with a catchment area of 172,854 habitants, located in the South Kivu Province of DRC, bordering with Rwanda and Burundi to the east (Bureau Central Zone Santé de Shabunda, personal information, 2013). The area has long been affected by conflict. MSF supports a general hospital run by the Ministry of Health in Shabunda town, a hospital centre in the village of Matili (around 30 km south of Shabunda) both providing secondary-level health care, and seven health centres (OPDs) providing primary-level health care to the whole catchment area.

The study took place in 2013, in the OPD of the general hospital of Shabunda and the OPD Mbangayo (also situated in Shabunda town) and in the households of the study population. Malaria activities are completely integrated in the hospitals and OPD activities. The study was conducted during November to coincide with the highest period of malaria incidence and therefore to reflect the level of adherence when the duration and quality of malaria care might vary because of the high caseload.

### Participants

Patient inclusion criteria were: age ≥1 year; a confirmed diagnosis via RDT (SD Bioline HRP2^®^) of uncomplicated falciparum malaria, with or without additional diagnoses; receipt of a course of FDC ASAQ during normal consultation hours in the MSF-supported OPDs; no previous participation of a household member in the study; and signed informed consent (adherence study) or verbal consent (exit interview). Patients were excluded from the adherence study if they were resident ≥30 min from the OPDs by foot or lived in Kitete, a “quartier” (quarter) of Shabunda town considered inaccessible due to security constraints. Kitete borders the river Ulindi, and rebel forces have their base on the other bank. Moreover, the airstrip, which was under fire 3 days before the start of the study, must be crossed to reach Kitete.

### Study procedure and data collection

All interviews were held either in one of the local languages (Swahili or Kirega) or in French. The study was supervised by the principal investigator and a field study supervisor who was fluent in all spoken languages. Prior to initiating the study, 2-days of training were given to all interviewers and data collectors. Extra attention was paid by the supervisors to discuss and agree on the same wording for all interviewers and data collectors while carrying out the interviews in the different local languages. With the help of role plays, all interviewers and data collectors had sufficient time to familiarize themselves with the questionnaires.

### Screening

The usual procedures of consultation, treatment prescription and dispensing in the MSF-supported OPDs were followed: patients received a health card and a malaria RDT if they were suspected of having malaria, defined as fever or history of fever in the previous 48 h; were prescribed FDC ASAQ in the case of a positive RDT and further drugs if necessary; took the first dose of FDC ASAQ under supervision at the pharmacy; and were given the second and third daily doses to take at home with intake instructions.

Before leaving the OPD, a short screening questionnaire was administered by three data collectors: a full description of all drugs prescribed was copied from each patient’s health card and their address recorded to enable subsequent location of their household. All patients, irrespective of diagnosis, received the screening questionnaire to avoid raising suspicion among health staff and patients/caretakers of patients about the specific purpose of the study. Health staff were informed that a study was taking place, but the exact purpose of the study was not revealed to avoid influencing routine prescribing habits.

### Adherence study

Patients prescribed FDC ASAQ were visited at home on the day after the last dose should have been consumed. If the visit could not be completed, the interviewer tried again the following day. No interviews were carried out at a later date. Patients needed to be at home to answer the questionnaire themselves. For children or patients not able to take FDC ASAQ on their own, the caretakers who gave them their treatment had to be present to answer the adherence questionnaire. Patients or caretakers who were not able to be located were classified as lost to follow-up and excluded from the study.

The semi-structured adherence questionnaire was developed in French. It included initial general questions about the patient and household: sex, age, education level of patient or caretaker, household composition, and occupation of head of household. A systematic account of how the tablets had been taken was recorded. Patients or caretakers were also asked to show the original co-formulated blister package of the FDC ASAQ; if it was still available any remaining tablets were counted. Finally, two questions on general malaria knowledge were asked. Five interviewers administered the adherence questionnaires at the home of the patients.

### Exit interviews with patients who received FDC ASAQ prior to leaving the OPDs

After all the screening questionnaires had been completed, exit interviews were carried out at the OPDs to help understand patient/caretaker attitudes towards FDC ASAQ prescription and to assess prescription quality (the information and instructions provided to the patient/caretaker and supervision of the first dose). All patients who received FDC ASAQ during normal consultation hours were eligible. They or their respective caretakers were interviewed upon exiting the OPD consecutively until the required number was achieved. Patients/caretakers were asked to describe how they were planning to give/take the FDC ASAQ, followed by a series of systematic questions for each treatment dose. Three interviewers (who also administered the screening questionnaires) conducted the exit interviews at each of the two OPDs.

### Definition of adherence

Since there is no standard definition for adherence we used definitions shown to be reliable in similar settings [25–29]. Patients were classified into three adherence categories (Table 1). Certain non-adherence: patients or caretakers who showed an FDC ASAQ co-formulated blister package still containing any tablets of FDC ASAQ at the home visit (certain incomplete intake). Probable non-adherence: patients who said that not all the tablets had been taken, but no package could be shown or it was empty (probable incomplete intake); or patients who said that not all the tablets had been taken according to the prescribed time schedule or dosage, but no package could be shown or it was empty (probable incorrect intake). Probable adherence: patients with a verbal account of complete treatment exactly following the treatment protocol and no package could be shown or it was empty (probable correct intake).

**Table 1:** Classification of adherence study patients by treatment adherence

Classification	Inclusions (n = 148)		
	n	%	95 % CI
Certain non-adherence (incomplete)	17	11.5	7–17
Probable non-adherence (probable)	20	13.5	8–19
Incomplete	8	5.4	3–10
Incorrect	12	8.1	4–13
Probable adherence	111	75.0	68–82
1st intake at OPD	74	50.0	42–58
1st intake at home	37	25.0	18–32

*95* *% CI* 95 % confidence interval, *OPD* outpatient department

### Sample size and sampling procedure

On the basis of malaria adherence studies in similar areas, adherence was conservatively estimated at 50 % [25–28]. With precision of 10 %, α-error of 5 %, and loss to follow-up or withdrawal of 20 %, the sample size required for the adherence study was 117. An equal number of patients was included in each OPD. Similar calculations resulted in a sample size of 117 patients for the exit interviews. Again, an equal number of patients was included in each OPD. Once the consultations of the day were finished in the OPDs, eligible patients were selected for the adherence study and assigned a permanent consecutive inclusion number. This sampling procedure was carried out in the two OPDs until the required number of patients/caretakers per OPD was achieved.

### Data management and analysis

Data were entered into EpiData 3.0 software (EpiData Association, Odense, Denmark). Data were cleaned by checking inconsistencies in data entry and responses. Data analysis was conducted using SPSS 10.0 for Windows (SPSS Inc., Chicago, USA). Baseline characteristics of patients/caretakers were described (mean age, male/female sex ratio, educational level). The proportions of probable/certain (non-) adherence and complete/incomplete/correct treatment intake were expressed with 95 % confidence intervals (95 % CIs). Risk factors for non-adherence such as age, education level and knowledge of malaria were analysed. Both *p* value and relative risks (with 95 % CI) were calculated where appropriate. The adherence and exit study populations were compared by comparison of proportions using the Chi squared test.

### Ethical considerations

The Ethics Review Board of MSF and the Ethics Committee of DRC granted ethical approval. Authorities at all health levels and heads of the different quarters in Shabunda town were informed via personal visits before the study started. Written consent was sought from patients or responsible caretakers of patients before the adherence questionnaire was administered. Verbal consent was sought from patients, or responsible caretakers participating in the exit interviews.

The adherence and exit questionnaires were anonymous. All data remained, throughout data entry and analysis, anonymous at the patient level. All participants received an explanation of the study purpose in the language they were most comfortable with—Swahili, Kirega, or French. Participation was voluntary and all participants were offered the opportunity to refuse participation at any time without penalty.

## Results

**Fig. 1:**
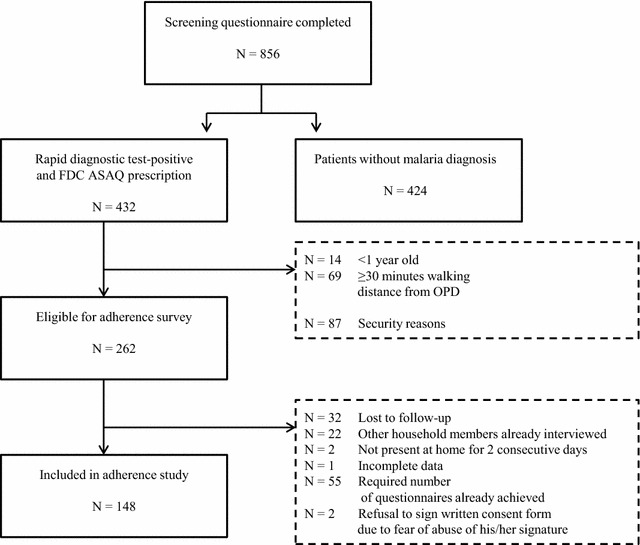
Study profile for FDC ASAQ adherence study Shabunda Health Zone, Democratic Republic of the Congo. *FDC ASAQ* fixed-dose combination artesunate–amodiaquine

Data were collected between November 13 and 21, 2013. In total, 856 patients were screened at the two OPDs. Of these, 432 received an FDC ASAQ prescription (50.5 %), 262 (60.6 %) of whom were eligible for inclusion in the adherence study; 148 patients/caretakers were finally included (Fig. 1). Of the 148 patients in the adherence study, 56 (37.8 %) had come without and 92 (37.8 %) with a caretaker to the OPDs. General characteristics of the adherence study population are shown in Table 2.

**Table 2:** General characteristics of adherence study population (patients and caretakers)

	Patient (n = 148)		Caretaker (n = 92)	
	n	%	n	%
Age classes (years)				
<5	42	28.4	–	–
≥5	106	71.6	92	100.0
Mean, median (minimum–maximum)	13.4, 10 (1–60)	34.4, 32 (13–68)		
Gender				
Male	62	41.9	26	28.3
Female	86	58.1	66	71.7
Highest education level				
Illiterate	7^a^	12.5	21	22.8
Primary	16^a^	28.6	23	25.0
Secondary	33^a^	58.9	43	46.7
Higher	–	–	5	5.5
Household size				
1–4 members	9	6.1		
5–15 members	114	77.0		
>15 members	25	16.9		
Mean household size (members)	10.6			
Household owns ≥1 insecticide-treated mosquito net	137	92.6		
Numbers of children <5 years per households				
0 children	14	9.5		
1 child	28	18.9		
2–4 children	91	61.5		
5–9 children	15	10.1		
Mean number of children <5 years per household	2.4			
Main 3 professions of heads of households				
Subsistence farmer	58	39.2		
Civil servant	15	10.1		
Teacher	13	8.8		

^a^7/56, 16/56 or 33/56 patients who came to the OPDs without a caretaker

### Patient adherence

Among the 148 patients interviewed, 11.5 % (17/148, 95 % CI 7–17) had one or more tablets left at the time of the home visit and were defined as certainly non-adherent; 13.5 % (20/148, 95 % CI 8–19) were defined as probably non-adherent, giving a total of 25.0 % (37/148, 95 % CI 18–32) non-adherence. Seventy-five percent (111/148, 95 % CI 68–82) were defined as probably adherent (Table 1). Adherence did not vary significantly between the under- and over-5 year age groups, with probable adherence of 61.9 % (26/42, 95 % CI 47–76) versus 80.2 % (85/106, 95 % CI 72–87), respectively (*p* = 0.19). Seventeen (11.5 %, 17/148) of the interviews at home were carried out 2 days after the last dose should have been consumed. No difference in adherence could be seen between the interviews carried out 1 or 2 days after the last dose should have been consumed.

### Reasons for incomplete, incorrect or correct intake

The two main reported reasons for incomplete intake were sickness after FDC ASAQ intake (28 %, 7/25) and no food/sugar available in the household for FDC ASAQ intake (20 %, 5/25) (Table 3); 70.6 % (18/25) of patients with incomplete intake omitted their last treatment dose. The main reason for incorrect intake was immediate vomiting after drug intake (66.7 %, 8/12), but otherwise malaria treatment had been taken correctly. The main reason stated for correct FDC ASAQ treatment intake was that the instructions given at the OPD had been followed (87.8 %, 65/74) (Table 3). For patients who had correctly taken their treatment, but taken the first dose at home, 48.6 % (18/37) reported not eating prior to the OPD visit (Table 3).

**Table 3:** Reasons for incomplete, incorrect or correct FDC ASAQ intake

	n = 149	%
Reasons given for incomplete intake (n = 25)		
Sickness after FDC ASAQ intake	7	28.0
No food/sugar available in the household for FDC ASAQ intake	5	20.0
Forgot to give/take FDC ASAQ	3	12.0
Reported that instructions at OPD were not understood	3	12.0
Patient felt better, no reason to continue with FDC ASAQ intake	3	12.0
Reported vomiting after FDC ASAQ intake	3	12.0
Could not find blister to finalize the FDC ASAQ intake	1	4.0
Reasons given for incorrect intake (n = 12)		
Reported vomiting after FDC ASAQ intake	8	66.7
Sickness after FDC ASAQ intake	2	16.7
Thought patient would get better faster	1	8.3
Reported that instructions at OPD were not understood	1	8.3
Reasons given for correct intake—first dose given at OPD (n = 74)		
Reported that correct intake instructions were given at OPD	65	87.8
Knew how to take/give FDC ASAQ from previous treatment	6	8.1
Other reasons^a^	2	2.7
No reasons given	1	1.4
Reasons given for correct intake—first dose taken at home (n = 37)		
Patient had not eaten prior to OPD visit	18	48.6
OPD told patient/caretaker to take/give first dose at home	7	18.9
No reasons given	9	24.4
Other reasons^b^	3	8.1

*FDC ASAQ* fixed-dose combination artesunate–amodiaquine, *OPD* outpatient department

^a^Other reasons were: (i) caretaker did everything to ensure that the child gets better; and (ii) the MSF health educator told patient the correct intake of the treatment

^b^Other reasons were: (i) health staff at OPD were very busy; (ii) the teacher and not a caretaker brought the child to the OPD; and (iii) caretaker reported that health staff did not want to give first dose at the OPD, as the patient’s sister was known to have reacted previously adversely

### Assessment of possible risk factors and knowledge on malaria

None of the risk factors such as age, education level, and knowledge of malaria were associated with increased risk of non-adherence (n = 37). When adherence study patients or their respective caretakers were asked about the cause of malaria, 60.8 % (90/148, 95 % CI 52.8–68.4) mentioned mosquito bites and 39.2 % (58/148, 95 % CI 31.6–47.2) claimed not to know the cause of malaria or mentioned a wrong cause. In total, 79.1 % (117/148, 95 % CI 72.0–85.0) mentioned that sleeping under an insecticide-treated mosquito net is a way of preventing malaria and 20.9 % (31/148, 95 % CI 15.0–28.1) did not know any means of prevention or mentioned an incorrect means of prevention.

### General characteristics of the exit interview group

Exit interviews were carried out with 120 patients. The general characteristics of this group are shown in Table 4. The socio-demographic and clinical characteristics of this group did not differ from those of the adherence study (mean patient age, proportion of patients <5 years, gender of patients/caretakers, education level; all *p* > 0.05).

**Table 4:** General characteristics and understanding of anti-malarial treatment intake in the exit interview study population

	Patients (n = 120) [n (%)]	Caretakers (n = 92) [n (%)]
Age (years)		
<5	43 (35.8)	–
≥5	77 (64.2)	92 (100.0)
Mean, median (minimum–maximum) (years)	10, 5 (0–60)	28, 27 (10–68)
Gender		
Male	64 (53.3)	9 (9.8)
Female	56 (46.7)	83 (90.2)
Highest education level		
Illiterate	3/28^a^ (10.7)	17/91^b^ (18.7)
Primary level	12/28^a^ (42.9)	37/91^b^ (40.6)
Secondary level	12/28^a^ (42.9)	35/91^b^ (38.5)
Higher level	1/28^a^ (3.5)	2/91^b^ (2.2)

	n	[%]
Perception of disease (n = 120)		
Able to name disease as malaria	33	27.5
Able to name signs/symptoms of malaria	72	60.0
Disease and its signs/symptoms unknown *or* other symptoms mentioned	15	12.5
Perception of anti-malarial treatment (n = 120)		
Able to show FDC ASAQ as only malaria treatment	97	80.8
Able to show FDC ASAQ together with paracetamol	10	8.3
Shows only paracetamol	2	1.7
Unable to distinguish any given treatment	11	9.2

	n/N	[%]
Patient or caretaker of patient		
Was able to correctly repeat the instructions for FDC ASAQ intake	107/120	89.2
Errors made while repeating the instructions for FDC ASAQ intake	13/120	10.8
Second dose will be wrongly taken at first intake day in the evening	6/13	46.2
Split dose in half and take first half in the morning and second half in the evening	4/13	30.8
Unable to repeat instructions	2/13	15.4
Will start with FDC ASAQ the next day (first dose was not given at OPD)	1/13	7.7
Will continue with treatment although patient feels better the next day	114/120	95.0
OPD role		
Was asked in the OPD if she/he had understood instructions	87/120	72.5
Was asked to repeat instructions	39/120	32.5
Was given additional information related to FDC ASAQ	43/120	35.8

*FDC ASAQ* fixed-dose combination artesunate–amodiaquine, *OPD* outpatient department

^a^Number of patients who came to the OPDs without a caretaker

^b^1 missing value

### Understanding of FDC ASAQ intake in the exit interview group

Of the 120 patients in the exit interview group, 28 (23.3 %) had come without and 92 (76.7 %) with a caretaker to the OPDs. Of this group, 87.5 % (105/120) knew that they had malaria or could name the correct signs/symptoms of malaria. Eighty-nine percent (107/120) were able to identify the FDC ASAQ tablets (in combination with paracetamol) as anti-malarials among all the tablets given (Table 4). Eighty-nine percent (107/120) were able to correctly repeat the full FDC ASAQ intake instructions given to them at the OPDs. Of the 10.8 % (13/120) who were not able to correctly repeat these instructions, the two most common errors were to take the second dose in the evening of the first day of treatment intake (46.2 %, 6/13) or to split the dose in half and take the first half in the morning and the second half in the evening (30.8 %, 4/31). Ninety-five percent (114/120) said they would continue with the FDC ASAQ intake even if they/the patient felt better during the treatment period.

According to patients/caretakers, 72.5 % (87/120) of the health staff in the OPDs had asked them if they had understood the FDC ASAQ intake instructions. Only 32.5 % (39/120) were asked to repeat these instructions and 35.8 % (43/120) were given additional information by health staff related to FDC ASAQ. In most cases (72.1 %, 31/43) this advice was to take sugar water or eat before the FDC ASAQ intake.

## Discussion

The results of this study show that good adherence to the correct intake of FDC ASAQ is possible even in this remote, conflict-affected region. The high reported rate (75 %) of probable adherence in this rural area is encouraging and could help prolong the effectiveness of first-line malaria treatment regimens. However, there was a (non-significant) finding that patients younger than 5 years were less likely to be completely adherent (62 %), which is concerning since children are more likely to develop severe disease, increasing the risk of drug resistance.

When assessing the main reason for correct FDC ASAQ intake, almost all patients claimed they understood the instructions given to them at the OPDs. When assessing the exit interview group, an equally positive statement can be made for the quality of FDC ASAQ prescriptions and the instructions given at the OPDs: almost all patients were able to identify FDC ASAQ as the anti-malarial treatment amongst other concomitant treatments and could correctly repeat the instructions given to them in the OPDs. This shows much better quality of anti-malarial prescriptions than in a similar study conducted in The Gambia where only a third of caretakers were able to name the disease for which they had visited the health facility [30, 31].

ACT should ideally be prescribed in combination with paracetamol. Very positively, almost 96 % of patients had received a prescription for paracetamol. According to the home interviews, adherence to paracetamol was high, with more than 90 % of patients with a paracetamol prescription taking it with the FDC ASAQ.

Only 11.5 % of patients were certainly non-adherent with another 13.5 % meeting the criteria for probable non-adherence. Of the patients that were non-adherent (certain and probable), in two-thirds this was related to incomplete intake and in one-third to incorrect intake. The main reasons patients gave for incomplete intake was that the treatment made them feel sick or there was neither food nor sugar water at home. Two-thirds of the patients with incomplete intake did not take their third and last dose of FDC ASAQ. This result might be linked to the finding in the exit interviews that only a third of patients or caretakers were given any additional information related to FDC ASAQ. Of those who received additional information, none mentioned being told that the treatment may cause sickness and fatigue. The health staff placed emphasis on taking the treatment together with food or sugar water, which might have led to patients deciding to miss a dose rather than take it on an empty stomach. Around two-thirds of patients who took the drug incorrectly did so because they vomited immediately after intake. The remaining few patients with incorrect intake mostly took the second dose in the evening of the first day of treatment intake. These results matched the results of the exit interview group: only 10 % could not correctly repeat the instructions for FDC ASAQ intake, the most common error was to take the second dose in the evening of the first day.

None of the risk factors such as age, education level, and knowledge of malaria were associated with an increased risk for non-adherence. Nevertheless the study population seems to have a relatively high level of understanding of the disease, shown previously to be significantly linked to good adherence [32]. This could be linked to the slightly higher level of education in study population compared to the national average [33]. Two-thirds of adherence interviewees said they knew the cause of malaria and almost 80 % knew that sleeping under an insecticide-treated mosquito net is a means of prevention. In the exit interview group, 80 % of patients or caretakers could name malaria or knew the signs and symptoms.

Two different methods, considered to be complementary, were used to measure adherence in patients: the pill count and a systematic questionnaire. Biological assays may offer more precise adherence measurements [34–36]. They are, however, dependent on the availability of laboratory testing and, therefore, not practical in many resource-limited and unstable settings. Both the assessment methods we used have their limitations: the pill count is a more objective measurement; if the interviewers observe remaining tablets, they can be absolutely certain that the patient is non-adherent. The questionnaire gives more complete information, such as on timing of doses, but is subjective and not verifiable. By classifying patients as either certainly or probably non-adherent, the study took the limitations of both assessment methods into account. To reduce error, patients were asked non-judgmentally about medication-taking behaviours and the delay between treatment and questionnaire administration was minimized—none of the interviews were carried out later than 2 days after completing treatment [18]. Exit interviews were added to the study design to gain information on treatment attitudes and to assess prescription quality. Use of the three complementary methods strengthened the findings of the study.

One of the most difficult things to avoid in adherence studies is influencing health staff such that they change prescribing habits. It is also important not to alert patients that there might be a later check-up regarding the treatment in order to avoid good-will bias [12, 17, 37]. The following measures were taken to limit these sources of bias: the screening questionnaire was carried out for malaria and non-malaria patients attending the OPDs to avoid raising any suspicion among the health staff, patients or their caretakers. Informed written consent was sought only from those patients interviewed at home. The true nature of the study was not revealed to the OPD health staff at the time of the study. Exit interviews took place only after all the screening questionnaires had been completed so as not to influence anybody involved in the study.

Initially the authors’ wanted to include the hospital centre in the village of Matili, located roughly an hour’s car journey from Shabunda town, as a study site. However, due to the deteriorating security situation in the region, only Shabunda town health facilities were able to be included. As very remote areas are usually linked to lower adherence, lower adherence levels might have been found if the most remote areas of Shabunda Health Zone had been included.

Although more patients were eligible to participate in the study, the deteriorating security situation in the region meant that data collection needed to be completed as quickly as possible. Participant recruitment was therefore stopped once the calculated sample size had been reached.

In 2005, the first systematic review on malaria adherence concluded that there was insufficient information on this subject and research would benefit from of standardization of methodologies [38]. In 2014, the second systematic review on adherence to ACT treatment was published but only 37 studies were eligible for inclusion [17]. The lack of uniform methodology and definitions for adherence makes comparison between studies difficult. Only a few of the eligible 37 studies used detailed definitions of adherence that included doses, duration, and frequency and many revealed the purpose of the study and alerted the study population to home visits to assess treatment intake.

In the seven studies carried out on adherence to the ACT combination ASAQ in Africa between 2008 and 2012, adherence varied between 48 and 97 %. In three studies in which adherence was >90 % the purpose of the study was known both by health staff and the study population [39–41]. In two studies with adherences of 48 and 77 %, respectively, health staff and the study population were not aware that adherence would be assessed [28, 42]. In the only two studies that were carried out with FDC ASAQ, adherence was as high as 83 and 90 %. However, both studies were designed as effectiveness studies with adherence as a secondary objective and study patients were aware that adherence would be assessed [23, 24].

The adherence result of 75 % in this study is higher than in studies with a related design (conducted in routine health service delivery with patients/caregivers not informed about the aim in advance), methodology (self-reports from patients/caregivers in combination with pill counts), similar study areas (remote rural areas), and preconditions (anti-malarial treatment already implemented for some time). In Ethiopia, adherence was 39 %, in Zambia 40 %, in Sierra Leone 48 %, in South Sudan 53 %, in the Central African Republic 61 % and in Kenya 64 % [12, 25–27, 43, 44].

In the exit interview group, almost half the patients or their caretakers claimed not to have taken the first treatment dose under supervision in the OPDs. According to adherence interviews, the main reason was that the patients had not eaten prior to the OPD visit and therefore were told to take the first dose at home together with food. Given the high overall adherence in the study, the intake of the first dose under supervision does not seem to have a big influence on adherence and contradicts the results of previous studies where supervised intake of the first anti-malarial treatment was directly correlated with higher adherence [38, 42, 44].

Access to prompt and effective treatment is a cornerstone of the current malaria control strategy. A delay in starting appropriate treatment is a major contributor to malaria mortality. Many patients and especially children with suspected malaria, where medical services are not easily accessible, start treatment too late or do not receive it at all and die at home. Therefore, since 2004, WHO has recommended home- or community-based management of malaria by trained community health workers as one of the strategies for improving access to prompt and effective malaria case management [45]. However, crucial to the sustainable success of community-based malaria management is strengthening health system capacities to support supply, for training and close quality supervision, and foremost for appropriate treatment of referred cases [46, 47]. In a study carried out in Uganda, only one in five community health workers performed optimally [48]. If adequate resources for community-based malaria management are not given and close monitoring of activities cannot be guaranteed, it might be necessary to first increase the capacity for correct malaria case identification and management at the peripheral health centre level. The results of the study provide evidence that patient adherence to the correct intake of ACT and high-quality prescribing at health centre level is possible in this setting, despite being remote and largely unstable. High levels of adherence to ACT prescribed in health facilities might be a first step to the successful introduction of home- or community based management of malaria. High levels of resistance to older anti-malarials mean that ACT should remain the first-line treatment for malaria. The effectiveness of ACT depends on its efficacy and on adherence to treatment. Assessments of ACT adherence at the household and exit interviews at the health facility level should be used to inform operational programming in areas of high malaria-burden to maximise the therapeutic value of the treatment. Moreover, further ways to prevent and diagnose malaria need to be explored.

## Conclusions

To the authors’ knowledge, this is the first study to assess adherence to an FDC of ACT under real treatment conditions in a context of high instability. High quality prescribing of anti-malarials at health centre level and patient adherence to the correct intake of ACT were possible in this setting. Adherence to treatment regimens requires careful and constant monitoring, which might be better guaranteed at health centre rather than community level. It could, nevertheless, be a precondition to the successful introduction of home- or community based management of malaria.

## Abbreviations

ACT: artemisinin-based combination therapy
AQ: amodiaquine
AS: artesunate
DRC: Democratic Republic of the Congo
FDC: fixed-dose combination
MSF: Médecins Sans Frontières
OPD: outpatient department
RDT: rapid diagnostic test
SP: sulfadoxine–pyrimethamine
WHO: World Health Organization
95 % CI: 95 % confidence interval
